# Electrochemical Synthesis of Nanomaterials Using Deep Eutectic Solvents: A Comprehensive Review

**DOI:** 10.3390/nano16010015

**Published:** 2025-12-22

**Authors:** Ana T. S. C. Brandão, Sabrina State

**Affiliations:** 1 CIQUP/IMS—Chemistry Research Center, Faculty of Sciences, University of Porto, Rua do Campo Alegre 1021, 4169-007 Porto, Portugal; ana.brandao@fc.up.pt; 2 Faculty of Medical Engineering, National University for Science and Technology POLITEHNICA Bucharest, 1-7 Gheorghe Polizu Street, 011061 Bucharest, Romania; 3 National Institute for Research and Development in Microtechnologies—IMT Bucharest, 126a Erou Iancu Nicolae, 077190 Bucharest, Romania

**Keywords:** deep eutectic solvents, electrochemical synthesis, nanostructures, morphology control, nucleation mechanism, nanomaterials applications

## Abstract

Deep eutectic solvents (DES) have emerged as a versatile and sustainable medium for the green synthesis of nanomaterials, offering a viable alternative to conventional organic solvents and ionic liquids. Nanomaterials can be synthesised in DESs via multiple routes, including chemical reduction, solvothermal, and electrochemical methods. Among the different pathways, this review focuses on the electrochemical synthesis of nanomaterials in DESs, as it offers several advantages: low cost, scalability for large-scale production, and low-temperature processing. The size, shape, and morphology (e.g., nanoparticles, nanoflowers, nanowires) of the resulting nanostructures can be tuned by adjusting the concentration of the electroactive species, the applied potential, the current density, mechanical agitation, and the electrolyte temperature. The use of DES as an electrolytic medium represents an environmentally friendly alternative. From an electrochemical perspective, it exhibits high electrochemical stability, good solubility for a wide range of precursors, and a broad electrochemical window. Furthermore, their low surface tensions promote high nucleation rates, and their high ionic strengths induce structural effects such as templating, capping and stabilisation, that play a crucial role in controlling particle morphology, size distribution and aggregation. Despite significant progress, key challenges persist, including incomplete mechanistic understanding, limited recyclability, and difficulties in scaling up synthesis while maintaining structural precision. This review highlights recent advances in the development of metal, alloy, oxide, and carbon-based composite nanomaterials obtained by electrochemical routes from DESs, along with their applications.

## 1. Introduction

Nanomaterials, characterised by their dimensions typically ranging from 1 to 100 nm, exhibit unique physicochemical properties, such as high surface area-to-volume ratios, tunable optical characteristics and enhanced reactivity compared to their bulk counterparts [1]. These features make them invaluable across diverse fields, including catalysis [2], energy storage [3,4], biomedicine [5,6] and environmental remediation [7,8]. The synthesis environment plays a pivotal role in determining the size, morphology and stability of the resulting nanomaterials. Nanomaterial synthesis in aqueous solutions is a widely adopted method; however, it presents several challenges and limitations: (i) uncontrolled nucleation and particle growth, which leads to broad size distributions, (ii) agglomeration and poor colloidal stability, (iii) restricted solubility of metal and organic precursor and (iv) limited temperature ranges [9]. Therefore, these inherent drawbacks underscore the need for alternative solvent systems.

Ionic liquids (ILs) are salts composed entirely of ions and typically have melting points ≤ 100 °C, meaning they are liquid at or around ambient conditions. One of the first widely recognised examples was reported by Paul Walden in 1914, namely ethylammonium nitrate [EtNH_3_][NO_3_], which melts at approximately 12 °C [10]. Since then, extensive research has been conducted on this topic, and the number of published studies has increased exponentially [11]. There are three generations of ionic liquids, categorised by their chemical properties and structure [12,13]. The first generation of ILs consisted of large-volume species such as 1,3-dialkylimidazolium or 1-alkylpyridinium, with anions based on halogen aluminate. These types of ILs are sensitive to air and water. The second generation is characterised by its stability in air and water, and is mainly based on the dialkylimidazolium and alkylpyridinium cations combined with different anions, such as [BF_4_]^−^ or [PF_6_]^−^. The main drawbacks of this generation are its high toxicity and low biodegradability. Finally, the third generation of ILs consists of biodegradable and natural ions, characterised by their low toxicity. In general, ILs provide a versatile platform for combining organic heterocyclic cations (derivatives of imidazolium, pyrrolidinium, and piperidinium) with various organic (methyl sulfate, sulfonimide) or inorganic (halide, nitrate, acetate, dicyanamide) anions to tune solvent properties. Due to their negligible vapour pressure, ILs are effectively non-volatile and excellent solvents for a wide range of inorganic, organic, and polymeric substances. These features make ILs particularly attractive as reaction media for the synthesis of nanomaterials. Their low surface tension promotes rapid nucleation, thereby facilitating the formation of smaller nanoparticles. They can act simultaneously as both electron donors and spatial stabilisers, thereby inhibiting excessive nanoparticle growth and aggregation. Various reviews cover the synthesis of nanomaterials in ILs [14,15,16,17,18].

Deep eutectic solvents (DESs) were introduced as an alternative to ILs to overcome their limitations. Over the past few decades, DESs have attracted significant attention due to their favourable physicochemical properties and environmental benefits [19,20,21,22]. Although ILs and DESs shared specific characteristics, particularly in their physical behaviour, they are chemically distinct classes of liquids, with more differences than similarities. Unlike conventional ILs, DESs are typically formed through the complexation of a hydrogen bond donor (HBD) and a hydrogen bond acceptor (HBA), resulting in a eutectic mixture with a melting point much lower than that of the individual components. The deep melting-point depression in DESs arises from strong hydrogen-bonding and a significant increase in configurational entropy upon mixing [23]. The considerable advantage of DESs lies in their composition; they are generally non-toxic, biodegradable, inexpensive, and derived from cheap and sustainable compounds, thereby overcoming the drawbacks of ILs, as shown in Table 1 [20,24,25,26,27,28].

**Figure 1:**
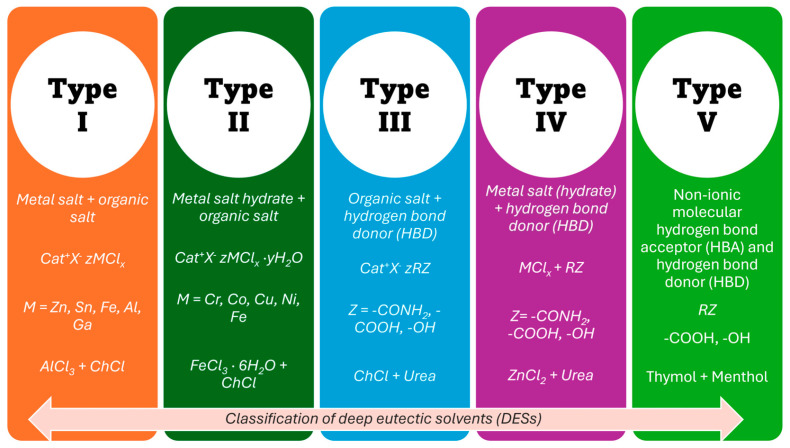
Classification of DESs [20,21,35,36].

The general formula of DESs is Cat^+^X^−^zY, where Cat^+^ is ammonium, phosphonium or sulfonium cation, X^−^ is the halide anion, usually Cl^−^, and Y refers to the Lewis or BrØnsted acid that makes a complex with the anionic species; z is associated with the number of Y molecules that are involved in complexation of anion. DESs can be categorised into five types depending on the complexing agent, as summarised in Figure 1 [28,29]. DES type I is formed by a mixture of ChCl (or other quaternary ammonium salts) with an anhydrous metallic salt, for instance ZnCl_2_ [33]. It is similar to the well-investigated chloroaluminate imidazolium melts or chlorozincate imidazolium melts, which are traditional ILs. When hydrated metal halides were used, the eutectic mixture was classified as type II [34]. It was found that a wide variety of mixtures of choline chloride with hydrated salts (CrCl_3_⋅6H_2_O, CaCl_2_⋅6H_2_O, CoCl_2_⋅6H_2_O, LiNO_3_⋅4H_2_O or Zn(NO_3_)_2_⋅4H_2_O) form these DESs.

In 2003, Abbott’s group reported the preparation of a novel, air- and water-stable mixture resulting from the combination of hydrogen bond donors (HBDs) with hydrogen bond acceptors (HBAs). It was observed that a mixture in a 1:2 molar ratio of choline chloride (Tm ~302 °C) with urea (Tm ~133 °C) results in a eutectic that is liquid at room temperature (Teutectic ~12 °C [37]). Then, many other binary mixtures of hydrogen bond acceptors (e.g., choline chloride, acetyl choline chloride, tetraethylammonium bromide, etc.) with hydrogen bond donors (e.g., urea, ethylene glycol, glycerol, carboxylic acids, polyols, etc.) were prepared, which motivated extensive research in the field that we know today as ‘Deep Eutectic Solvents’ (DESs) [33,38]. These eutectics are classified as type III, and their properties vary depending on the type of quaternary ammonium salt and HBD used. The number of possible combinations of HBA and HBD that can form eutectic mixtures is exceptionally high, and many have been reported to date.

Type IV DESs result from the combination of AlCl_3_ or hydrated salts of Cr and Zn with organic compounds as hydrogen bond donors (urea, ethylene glycol, 1,6-hexanediol and acetamide) [19,39,40]. Finally, Type V DESs represent the latest generation of eutectic systems, formed by a combination of non-ionic HBA and HBD components [39,40]. They are composed entirely of neutral molecular components, many of which are found in plants, such as natural monoterpenoids, including thymol, menthol, cineole, and carvone [41]. Abranches et al. proposed the type V DES obtained by mixing thymol and menthol in a 1:1 molar ratio [42]. They exhibit lower viscosity than other types of DESs, have low vapour pressure, and are chloride-free. Type V DESs, with different degrees of hydrophobicity, can be obtained since a large number of non-ionic substances are available. However, this new class of DESs is not as widely accepted as the others.

Within this broad classification, Natural Deep Eutectic Solvents (NADES) and Therapeutic Deep Eutectic Solvents (THEDES) have emerged as two significant subclasses with specific biomedical and environmental relevance. NADES are composed of naturally occurring components, such as amino acids, sugars, organic acids, and polyols, that mimic the intracellular environment, providing high biocompatibility and biodegradability [41]. They are promising green solvents for extractions and chromatographic media, showing potential applications in the pharmaceutical, cosmetic, and food industries [43,44]. On the other hand, THEDES are specifically designed to have one of the DES components as an active pharmaceutical ingredient, such as ibuprofen, acetylsalicylic acid, or DL-menthol. These solvent systems function as both drug carriers and active therapeutic agents, offering novel routes for drug formulation and delivery [45,46].

Regarding nanomaterial synthesis, as with ILs, DESs exhibit low surface tensions, which promote high nucleation rates. Additionally, the high ionic strength can induce structural effects, such as templating, capping and stabilisation, which help control particle morphology and prevent agglomeration [16]. Beyond serving as a solvent or structural template, the constituent species of DES can also act as reactive reagents, participating directly in the formation or functionalization of nanomaterials [47]. DESs have arisen as versatile and sustainable media for the synthesis of nanomaterials via multiple routes, including chemical reduction, solvothermal, and electrochemical methods. In chemical reduction, DESs can act as both reaction media and reducing agents, promoting the synthesis of nanostructures without the need for toxic reagents or stabilisers [48]. DES-solvothermal synthesis offers tunability over the size, shape and crystalline phase of the resulting materials [49,50]. In this process, DES acts as a latent supramolecular catalyst and a structure-directing agent [51]. Compared to conventional solvothermal methods, the use of DES represents a more environmentally sustainable approach, characterised by lower energy consumption and the elimination of volatile organic solvents [50]. However, among the preparation routes, electrochemical synthesis in DES has attracted significant attention as a clean, controllable method for producing nanomaterials with precise compositional and morphological features [21,35,36]. It offers several advantages, including low cost, scalability for large-scale production, and low-temperature processing [52,53]. The size, shape, and morphology of the resulting nanostructures can be tuned by adjusting the concentration of the electroactive species, the applied potential, the current density, mechanical agitation, and the electrolyte temperature. The use of DES as an electrolytic medium provides further benefits, including high electrochemical stability, excellent solubility for precursors, low toxicity, and a wide electrochemical window, which enable the synthesis of metals and alloys that are otherwise challenging to prepare in aqueous systems [28,36,54,55,56].

Several comprehensive reviews in the literature have addressed the electrodeposition of metallic coatings and alloys from DESs, and nanostructured electrochemical coatings are therefore not included in the present review [11,21,36,57,58]. Broader overviews of nanomaterial synthesis in DES-based systems are also available [35,48,59,60]. In contrast, this work focuses specifically on the electrochemical synthesis of nanostructures (e.g., nanoparticles, nanoclusters, nanoflowers, etc.) prepared from DESs, including metals, alloys, metal oxides, and carbon-based composites, and discusses their structural characteristics and applications.

## 2. Metal-Based Nanomaterials

Electrochemical synthesis in DESs has been proven particularly effective for preparing metal-based nanomaterials, owing to its ability to finely control nucleation and growth kinetics. The combination of high ionic strength, wide electrochemical window, and tunable solvent composition enables the formation of nanostructures with precise morphology, crystallinity, and composition. This section reviews recent progress in the electrochemical fabrication of metallic nanostructures, ranging from noble metals to transition metals, with a focus on how the composition of DES and electrochemical parameters govern particle size, shape, and functional performance.

### 2.1. Noble Metallic Nanostructures: Au, Ag, Pt, Pd and Ru

#### 2.1.1. Gold Nanostructures

**Figure 2:**
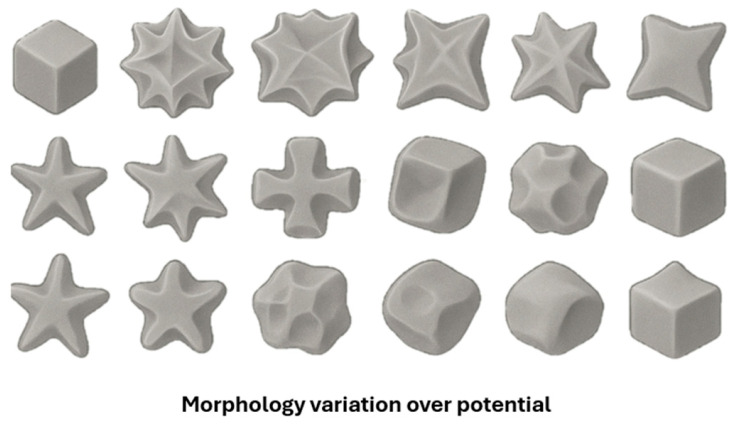
Schematic representations of Au NCs electrodeposited at a series of potentials. OpenAI (https://chatgpt.com/ (28 October 2025)), 2025.

Gold nanoflowers (Au NFs) and nanocrystals (Au NCa) have been successfully obtained from ChCl: Urea (1:2 molar ratio) using electrochemical routes. Nguyen et al. reported the electrochemical synthesis of Au NFs on a glassy carbon (GC) electrode by cyclic voltammetry (CV) within the potential range from −0.6 V to + 0.8 V (vs. Ag/AgCl) at a scan rate of 50 mV/s for 15 cycles using a DES based on ChCl: Urea with 5 mL of 50 mM HAuCl_4_ as electrolyte. The density and size uniformity of the AuNFs increased with increasing electrolyte temperature (40, 50, and 60 °C) due to enhanced reduction rates and accelerated nucleation at higher temperatures. The Au NFs-modified FC electrode exhibited remarkable performance as a label-free electrochemical DNA sensor for *M. tuberculosis* detection, achieving a sensitivity of 294.9 Ω nM^−1^ cm^−2^ and 218 µA nM^−1^ cm^−2^, and a limit of detection (LOD) of 10^−9^ M. The results demonstrate that DES is an environmentally friendly, low-cost, chemically stable, and biocompatible solvent in nanomaterials synthesis [61]. Wei et al. investigated the overpotential-dependent shape evolution of gold nanocrystals (Au NCs) electrodeposited on a GC electrode from a ChCl: urea DES electrolyte containing 24.28 mM HAuCl_4_. The potential ranged from −0.5 V to −0.95 V (vs. a Pt quasi-reference electrode). It led to a morphological evolution sequence from concave rhombic dodecahedra (RD) to concave cubes, octopods, cuboctahedral boxes, and finally to hollow octahedra (OH). This study demonstrated that urea molecules play a key role in shaping Au NCs, as they preferentially adsorb on the faces of seeds, blocking their growth. The morphology-dependent electrocatalytic performance of these Au NCs was evaluated for D-glucose electrooxidation, where concave rhombic dodecahedra (RDs) and concave cubes exhibited superior activity at lower potentials. In comparison, octopods and hollow octahedra became more efficient at higher potentials. In this work, the potential of DES is demonstrated as a powerful medium for shape-directed, surfactant-free nanocrystal synthesis [62]. Figure 2 presents a schematic representation of the shape evolution in response to potential change.

#### 2.1.2. Silver Nanostructures

J. A. Hammons et al. examined the nucleation and interfacial growth dynamics of Ag NPs on carbon electrodes in a DES electrolyte based on a mixture of ChCl and Ethylene Glycol, combining in situ grazing transmission small-angle X-ray scattering with simultaneous voltammetry and electrochemical impedance spectroscopy. This study provided nanoscale insights into the aggregation and coalescence of Ag NPs, multilayer perturbation induced by non-aggregated Ag NPs, and the stepwise depletion and transport of dissolved Ag species from the electrode surface. Both aggregation and silver ion depletion occurred slowly (~2 h), attributed to the high viscosity of DES, since both ion and particle transport are significantly impeded compared to water-based electrolytes [62]. Cojocaru et al. reported the pulsed reverse electrochemical synthesis of AgNPs at room temperature from a ChCl: Glycerol mixture using two silver plates as electrodes. The electrochemical synthesis was performed in the absence and presence of a capping agent, namely poly(N-vinyl pyrrolidone) (PVP). The nanoparticles range in size from 60 to 200 nm, as determined by Dynamic Light Scattering (DLS) measurements. It has been shown that the addition of PVP reduces NP size [63]. More recently, Lu et al. developed a green and efficient route for electrodepositing AgNPs in a ternary DES composed of ChCh:Urea: Glycerol (1:1:1 molar ratio) and explored its use as an electrochemical sensor for nitrofurazone detection. Ag was deposited on screen-printed carbon electrodes (SPCE) without additives and in the presence of hexadecyltrimethylammonium bromide (CTAB) and sodium dodecyl sulfate as surfactants (SDS). The incorporation of SDS into DES yielded Ag particles with a homogeneous distribution, demonstrating excellent analytical performance for nitrofurazone detection. A linear range was achieved from 0.66 µM to 47.62 µM and from 47.62 µM to 930 µM, with a limit of detection (LOD) of 0.37 µM. When applied to aquaculture water samples, the sensor achieved high recovery rates [64].

Moraes-Gil et al. reported the decoration of TiO_2_ nanotubes with noble metallic nanoparticles, namely Au and Ag. TiO_2_ nanotubes were anodically formed using aqueous electrolytes, while their decoration with Au and Ag NPs was performed by electrodeposition from ChCl:Urea (1:2 molar ratio) containing 5.0 mM HAuCl_4_ or 5.0 mM AgCl, respectively. The current-time transients indicated that the nanoparticle formation followed 3D nucleation and diffusion-controlled growth. The scanning electron microscopy (SEM) analysis revealed that the metallic nanoparticles are quasi-spherical, with an average diameter of 100 nm, and are distributed along the nanotube walls [65].

#### 2.1.3. Platinum Nanostructures

Platinum nanoflowers (Pt NFs) were successfully obtained using a DES based on ChCl: Urea. The morphology of the electrochemically synthesised Pt NFs was found to be highly dependent on the deposition parameters, particularly the precursor concentration, potential window, and number of cyclic voltammetry scans. At low concentrations of H_2_PtCl_6_, it was difficult to form Pt NFs with sharp, well-defined petals, leading instead to quasi-spherical aggregates. The number of CV cycles was optimised to 80, since fewer cycles produced irregular nanoparticles, while beyond 100 cycles, excessive aggregation and structural collapse occurred. The electrodeposition potential also played a crucial role in controlling the nanostructure’s morphology. The electrodeposition was performed at applied potential between −0.95 V, −0.90 V and −0.80 V. Notably, distinct flower-like nanostructures only appeared at −0.8 V. Both CV and constant potential deposition successfully produced Pt NFs with an average size of approximately 200 nm, demonstrating that shape and size can be finely tuned by modulating electrochemical parameters. Furthermore, the electrochemical testing revealed that the synthesised Pt NFs display significantly enhanced electrocatalytic activity and stability toward ethanol oxidation compared to the commercial Pt black catalyst [66].

**Figure 3:**
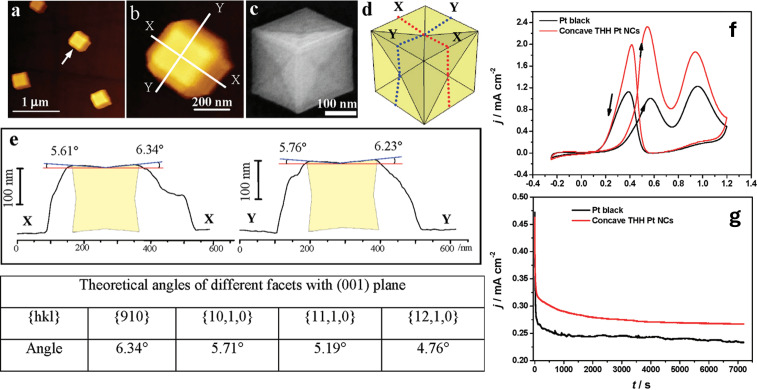
(**a**) Typical AFM images of concave THH Pt NCs in Figure 4a. (**b**) Magnified AFM image of concave THH Pt NCs indicated by the white arrow in (**a**). (**c**) SEM and (**d**) model images of a concave THH Pt NCs. (**e**) Cross-sectional profiles through white lines XX and YY in image (**b**), showing the XX and YY cross-sectional surfaces and the angles between concave facets and the (001) plane. (**f**) Cyclic voltammograms (50 mV s^−1^) and (**g**) chronoamperometric curves, measured at 0.45 V (vs. SCE), of ethanol oxidation on concave THH Pt NCs (red line) and commercial Pt black catalyst (black line) in 0.1 M ethanol + 0.1 M HClO_4_ solution. Reproduced with permission of Wei et al. [67]. Copyright © 2012, American Chemical Society.

The same group introduced a pioneering approach for the synthesis of monodispersed concave tetrahexahedral Pt nanocrystals (THH Pt NCs) using a GC electrode with ChCl and urea (see Figure 3).

To induce anisotropic crystal growth and achieve a concave morphology, a square-wave potential was applied with a lower potential limit of −1.30 V and an upper possible limit of −0.30 V for different deposition times of 20, 40, 60, and 80 min. The resulting Pt NCs exhibit well-defined concave THH geometries bounded by high-index {910} and vicinal facets, which were maintained across all deposition durations. However, the particle size increased progressively, from approximately 62.5 nm to 370 nm, as the deposition time increased. When evaluated as an electrocatalyst for ethanol electrooxidation, the THH Pt NCs displayed superior catalytic activity and long-term stability compared to commercial Pt black. The performance was attributed to the high-energy surface, which has a high density of active sites for ethanol adsorption and C-C bond cleavage [67].

#### 2.1.4. Palladium Nanostructures

**Figure 4:**
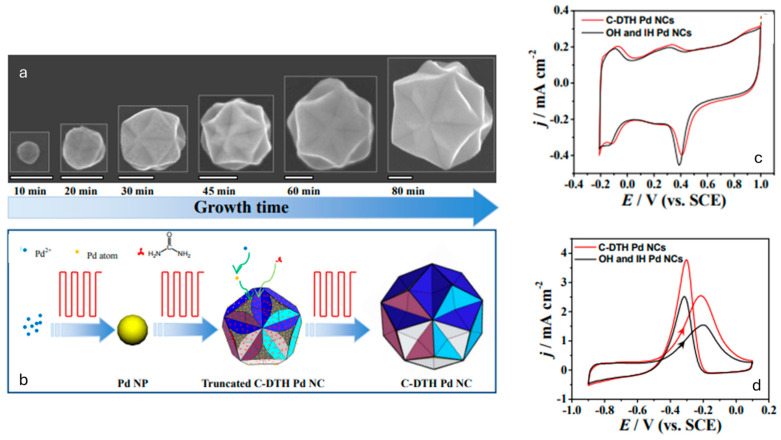
(**a**) SEM images of Pd NPs electrodeposited on GCE for different times, showing the growth process of C-DTH Pd NCs. Scale bars are 100 nm. (**b**) Schematic illustration of the proposed growth mechanism of C-DTH Pd NCs. Cyclic voltammograms recorded with C-DTH Pd NCs (red line) and a mixture of OH and IH Pd NCs (black line) in 0.1 M HClO4 solution (**c**) and 0.1 M ethanol + 0.1 M NaOH solution (**d**), respectively. Cyclic voltammograms were recorded at room temperature and a scan rate of 50 mV s^−1^. Reproduced with permission of Wei et al. [68]. Copyright © 2012, American Chemical Society.

Regarding Pd nanostructures, Wei et al. applied the square-wave potential method for the synthesis of concave-disdyakis triacontahedral palladium nanocrystals (C-DTH Pd NCs) from a ChCl:urea mixture (see Figure 4). The adsorption of urea has been shown to play a crucial role in determining the morphology of Pd nanostructures. Their shape can be electrochemically controlled, and perfect C-DTH Pd NCs are obtained when the EU is set at 0.05 V, and the EL is between −0.40 V and −0.50 V. The morphology of the nanostructures is influenced by the adsorbed urea formed at EU and the growth at EL. The C-DTH Pd NCs were found to exhibit improved catalytic activity for ethanol electrooxidation in alkaline media compared to the mixed morphologies obtained electrochemically at other EU and EL parameters [68].

Hammons et al. investigated the electrochemical synthesis, assembly, and solvent-particle interactions of Pd nanoparticles electrodeposited from a ChCl:urea (1:2) solution. The study employed a combination of in situ electrochemical techniques and ex situ imaging to elucidate how the DES composition and electrochemical conditions govern the formation of Pd nanoparticles. The authors demonstrated that DES stabilised the electrodeposited Pd NPs and that these particles self-assembled into extended layers rich in adsorbed anionic species. Electrodeposition was performed at two temperatures: 32.5 °C and 44.5 °C. Larger particles with a diameter of 20 nm were obtained at 44.5 °C, while smaller particles (0 nm) were obtained at lower temperatures [69].

Additionally, Juarez Marmolejo et al. conducted a detailed mechanistic and kinetic study of the electrochemical formation of Pd NPs on a GC electrode from a ChCl:urea (1:2) solution at 343 K. The evaluation of the current-time transient curves showed that the electrodeposition of Pd NPs followed a 3D nucleation-and-diffusion growth mechanism, accompanied by the simultaneous reduction of residual water at the growing Pd NP surface. The average particle size is ~32 nm or 16 nm, depending on the applied overpotential, −0.4 V and −0.54 V, respectively. Interestingly, the X-ray photoelectron spectroscopy (XPS) revealed a core–shell structure composed of metallic Pd^0^ cores surrounded by a thin Pd(OH)_2_ shell [70]. In a following works, the authors investigated the use of the deposited Pd@Pd(OH)_2_ core–shell nanoparticles for the electrocatalytic activity for methanol oxidation reaction in alkaline showing, indicating a mass activity of 2370 ± 450 mA mgPd^−1^ at the peak potential [71] and for electrocatalytic oxidation of formic acid from aqueous electrolytic bath with a mass activity of 5085 ± 129 mA mgPd ^−1^ [33,72].

Espino Lopez et al. reported the electrochemical synthesis of Pd NPs on a GC electrode from a DES based on ChCl:Urea at 298 K. The current-time transients revealed that Pd NPs form via 3D nucleation with diffusion-controlled growth, yielding particles with a size of 41 ± 5 nm. The XPS analysis showed that the Pd NPs are primarily composed of Pd(0) and contain a small amount of PdO or PdO_2_. The Pd NPs-modified electrode exhibited good electrocatalytic activity toward the formic acid oxidation reaction, revealing a mass activity of (4200 ± 100) mA mgPd^−1^ at the peak potential [73].

### 2.2. Other Metallic Nanostructures

#### 2.2.1. Nickel Nanostructures

**Figure 5:**
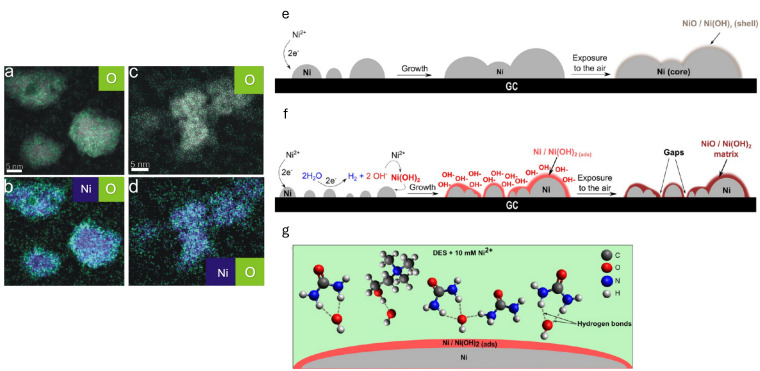
EDX map results for Ni deposited for 60 s at (**a**,**b**) E = −0.9 V and (**c**,**d**) E = −1.1 V. (**a**,**c**) The overlay between the HAADF image and O map. (**b**,**d**) The overlay between the Ni map and the O map. Scheme of the electrochemical processes occurring at (**e**) R1 and (**f**) R2. (**g**) Hydrogen bonds formed between hydroxides and DES components (urea and choline chloride). Reproduced with permission of Cherigui et al. [74]. Copyright © 2017, American Chemical Society.

Nickel nanoparticles (Ni NPs) with a dense nanostructured morphology were electrochemically synthesised on glassy carbon substrates using ChCl:Urea eutectic mixtures. The electrodeposition was performed under controlled potential conditions. The XPS analysis revealed that the nanoparticles possess a metallic Ni core and a thin layer of nickel oxide and/or hydroxide. The authors state that at a more negative potential, i.e., E = −1.1 V (vs. Ag QRE), the trace water in DES reduces to H_2_ or OH^−^ and can react with the nickel ions at the interface, leading to the formation of a precipitate of nickel hydroxide (see Figure 5). Furthermore, hydrogen bonding between the hydroxide species and the DES components (choline and urea) inhibits NPs growth [74]. Similar results were also reported by González et al. [75].

#### 2.2.2. Copper Nanostructures

Wang et al. reported, for the first time, the electrochemical preparation of copper nanoparticles (Cu NPs) using a DES electrolyte. The electrodeposition was performed on a stainless-steel sheet from an electrolyte containing chloroform (ChCl) and urea, with dissolved copper(I) oxide (Cu_2_O) as the reducing agent. Cu NPs with regularly equiaxed polyhedral shapes were obtained at constant currents of 2.5 V and 2.2 V, applied for 2 h. The TEM analysis revealed that at higher potentials, the Cu NPs are smaller than at lower potentials, specifically 28 ± 7 nm (2.5 V) vs. 57 ± 6 nm (2.2 V). At higher potential, the nucleation rate is faster than the growth rate of the nuclei, leading to smaller NPs sizes [76]. The authors continued their investigation, and Cu NPs were electrodeposited on nickel foils from Cu(I) (20 mM)/ChCl:Urea at 303 K by potentiostatic deposition (Q = 10 mC/cm^2^) at different potentials: −0.60 V, −0.70 V, −0.75 V and −0.80 V. At −0.60 V the Cu NPs are large and randomly distributed with diameters ranging from 100 to 160 nm. As the potential is shifted more negatively, the size of the Cu NPs decreases to 36 nm, and the number density increases progressively. The temperature also influences the size of the Cu NPs. An increase in the temperature from 303 K to 333 K is associated with a rapid growth in the size of the Cu NPs from 78 to 270 nm. The nucleation mechanism depends on electrolyte temperature, transitioning from progressive to instantaneous as the temperature increases [77].

Phuong et al. investigated the nucleation and growth mechanism of Cu NPs on a GC electrode from a ChCl:urea solution containing CuCl_2_·2H_2_O as a source of copper ions. The study focuses on the early stages of electrodeposition using cyclic voltammetry and chronoamperometry. The resulting Cu NPs were uniformly distributed and adherent to the substrate, as confirmed by SEM and EDX analyses. The authors observed a colour change in the copper electrolyte solution from blue to green upon heating, which was investigated using UV-Vis spectroscopy and attributed to the formation of a copper-water chloride complex. The CV and CA indicated that Cu NPs deposition can be performed potentiostatically, and the model is based on 3D nucleation and diffusion-controlled growth and adsorption [78].

Copper nanowires (Cu NWs) with diameters of 110–140 nm have been obtained electrochemically on poly carbonate membrane from ChCl:Ehylene glycol solution containing CuCl_2_ [79]. The electrodeposition was performed at constant potentials of −0.4 V and −0.44 V. However, DES’s higher viscosity than aqueous electrolytes may hinder ion diffusion into the membrane pores. To facilitate electrolyte access, higher working temperatures are recommended.

#### 2.2.3. Iron Nanostructures

Pardavé et al. reported the electrochemical synthesis of iron nanoparticles (Fe NPs) on Highly Oriented Pyrolytic Graphite electrode (HOPG) from a DES based on ChCl:Urea containing 50 mM FeCl_3_. The study investigated the electrodeposition mechanism using CV and CA. The electrodeposition of Fe NPs involved first the adsorption, followed by 3D nucleation with diffusion-controlled growth, which led to metallic iron formation, and lastly, the reduction of the residual water from DES, producing oxides on the surface of metallic nuclei, namely a mixture of FeO, Fe_2_O_3_, and Fe(OH)_3_. The nanoparticles are obtained by electrodeposition at −1.08 V for 30 s and exhibit a cauliflower-like morphology. The average size of the Fe NPs is 60 ± 8 nm; however, if electrodeposition is performed under forced convection, the particle density increases while their average diameter decreases [80].

#### 2.2.4. Bismuth Nanostructures

Bismuth nanowires (Bi NWs) were electrochemically synthesised without using any templates from ChCl:oxalic acid (1:1 molar ratio). The synthesis was performed on a metallic Cu substrate, using a solution containing BiCl_3_ as the electrolyte, ethylene glycol as the reducing agent, and polyvinyl pyrrolidone (PVP) to direct the growth of 1D structures. Bi NWs were formed under galvanostatic conditions at different current densities from 20 to 50 mA/cm^2^ during 30 to 60 min. The nanowires have a diameter between 100 and 120 nm and a length of 1–1.5 µm. The increase in the current density results in the formation of more 1D structures on the cathode without influencing morphology. The material was used as a sensor for H_2_O_2_ detection, with a linear range of 100 µM to 2.25 mM and a LOD of 33.3 µM [81].

### 2.3. Key Conclusions for Metallic Nanostructures

Electrochemical synthesis in DESs enables highly controlled fabrication of metallic nanostructures with complex, tunable morphologies, owing to DESs’ unique physicochemical properties. These features control nucleation and growth, allowing the formation of Au, Ag, Pt, Pd, Ni, Cu, Fe, and Bi nanostructures with shapes ranging from nanoflowers and concave polyhedra to hollow octahedra and 1D nanowires. Electrochemical parameters, such as potential, overpotential, temperature, precursor concentration, and the number of CV scans, among others, strongly influence morphology evolution. Due to the high viscosity of DES, its slow ion and particle transport promotes uniform growth and suppresses uncontrolled aggregation, yielding nanostructures with high-index facets and abundant active sites. As a result, the metallic nanostructures synthesised in DES exhibit exceptional electrocatalytic and sensing performance, frequently surpassing commercial electrocatalysts in reactions such as glucose, methanol, ethanol, and formic acid oxidation, and in electrochemical detection of DNA, nitrofurazone, and H_2_O_2_. Therefore, DESs represent a powerful, versatile and green platform for tailoring metallic nanostructures with application in diverse fields.

## 3. Alloys Nanostructures

### 3.1. Nobel Metals Alloys: Au-Pt, Cu-Ag, Pd-Ag

Bimetallic nanostructures combine the surface, electronic, and geometric effects of their constituent metals, often yielding catalytic performance that surpasses that of their monometallic counterparts. DESs have recently emerged as versatile, environmentally friendly electrolytes for the electrodeposition of noble-metal and noble-noble-metal alloys, enabling morphology control, enhanced surface area, and clean synthesis without surfactants. Representative examples include AuPt nanoflowers [82], Cu-Ag nanostructures [83] and Pd-Ag nanoparticles [84].

#### 3.1.1. AuPt Nanoflowers

**Figure 6:**
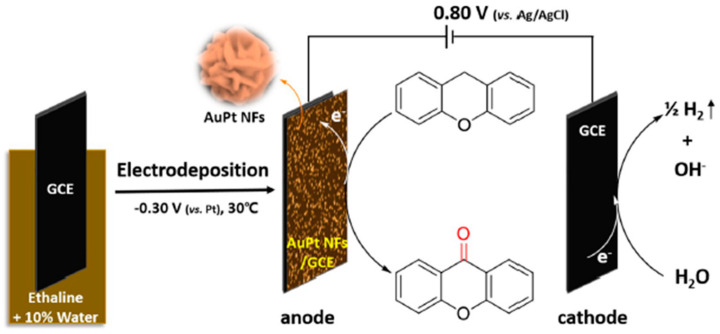
Scheme of the preparation of AuPt NFs and the electrochemical synthesis of XO from XT. Creative Commons CC BY licence [82].

AuPt alloy nanoflowers (NFs) were electrodeposited in ethaline DES (ChCl-ethylene glycol) with 10% water at −0.30 V vs. Pt and 30 °C, by Li et al. [82]. The moderate water content decreased viscosity, enabling deposition at low potential/temperature. The resulting flower-like nanostructures (~500 nm) displayed homogeneous Au-Pt alloying, confirmed by XRD, EDS, and XPS. Importantly, electrodes modified with AuPt NFs served directly as anodic catalysts for the electro-oxidation of xanthene to xanthone, achieving high yields under mild, oxidant-free conditions, as presented in Figure 6. This highlights the dual role of DES as both solvent and shape-directing agent, and the potential of DES-prepared bimetallic nanostructures for electroorganic synthesis.

#### 3.1.2. Cu-Ag Nanostructures

In a complementary study, Cu-Ag nanostructures were electrodeposited from a ChCl-urea DES at 70 °C, with varying Cu: bath ratios (1:1, 3:1, 6:1) and controlled applied potentials [83]. Morphology was highly sensitive to deposition rate. At moderate deposition (−0.65 V vs. Ag/AgCl), rounded porous nanoparticles (200–500 nm) formed, sometimes with unstable spikes. At a faster deposition rate (−0.75 V), stable, spiked nanostructures (200–300 nm) were consistently obtained. EDS and XPS confirmed homogeneous alloying, with Cu: Ag ratios matching the bath composition. Importantly, Cu-rich films were more stable and adherent, whereas Ag-rich deposits were fragile. Pb UPD analysis indicated significant increases in roughness factor and ECSA, confirming the catalytic relevance of these nanostructures for CO/CO_2_ reduction and oxygen reduction.

#### 3.1.3. Pd-Ag Alloy Nanoparticles

**Figure 7:**
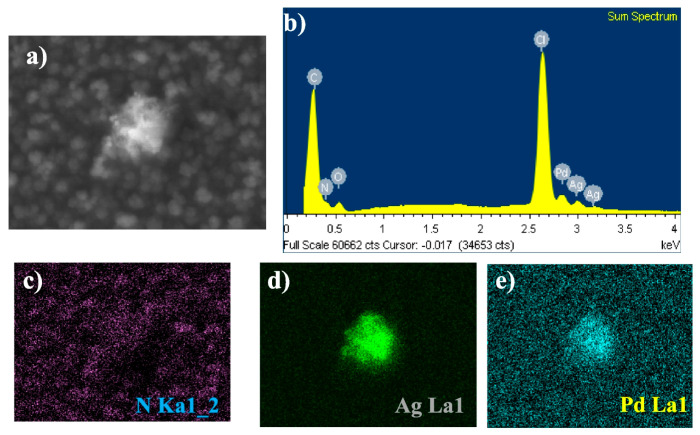
(**a**) SEM image of one of the Pd–Ag ANPs, (**b**) EDX spectrum and elemental mapping of (**c**) N Ka1, (**d**) Ag La1, and (**e**) Pd La1. Creative Commons Attribution 4.0 International License [84].

Ávalos-Huarte et al. synthesised Pd-Ag alloy nanoparticles (Pd-Ag NPs) in choline chloride-ethylene glycol mixture on both bare glassy carbon electrodes (GCE) and polypyrrole-modified GCEs (GCE/Ppy) [84], with the morphological characterisation presented in Figure 7.

Electrochemical analysis (CV, CA) revealed that Pd-Ag nucleation follows a multiple 3D nucleation and diffusion-controlled growth process, coupled with adsorption phenomena and double-layer charging. On bare GCE, two cathodic peaks were observed, corresponding to Ag and Pd reduction events. In contrast, on Ppy-modified GCE, only a single cathodic peak appeared, suggesting modified nucleation kinetics in the presence of the conductive polymer. Potentiostatic transients confirmed that alloy deposition was diffusion-limited and well-described by nucleation-growth models. The nucleation frequency increased by ~100 times on GCE/Ppy compared to bare GCE, underscoring the critical role of the conducting polymer in providing abundant nucleation sites and enhancing electron transfer. The morphology varies depending on the substrate used for synthesis. On bare GCE, Pd-Ag ANPs (~60 nm) were observed alongside larger quasispheroidal aggregates (~600 nm), while on GCE/Ppy only uniformly distributed ANPs (~50 nm) were obtained, with no aggregates. XRD analysis revealed the formation of an alloy with a slight lattice expansion resulting from Ag incorporation into Pd, confirming homogeneous substitutional alloying. XPS/EDX analysis confirmed a Pd-Ag composition of ~25% Pd and 75% Ag, closely matching the bath stoichiometry, with a homogeneous elemental distribution.

**Figure 8:**
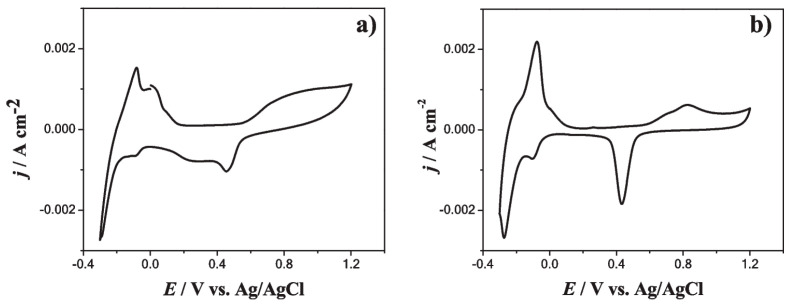
Experimental CVs recorded in the system: (**a**) GCE/PdAgNPs/0.1 M HClO_4_ and (**b**) GCE/Ppy/PdAgNPs/0.1 M HClO_4_ at 20 mVs^−1^ potential scan rate and 298 K. In both cases, the potential scan started at 0.2 V in the negative direction. Creative Commons Attribution 4.0 International License [84].

The electrocatalytic activity of Pd-Ag/GCE and Pd-Ag/GCE/Ppy electrodes was tested in acidic media. Both electrode types were stable in 0.1 M HClO_4_. However, the Pd-Ag/GCE/Ppy electrode exhibited vigorous mass activity toward formic acid oxidation, highlighting its promise for fuel cell applications, hydrogen production/storage, and electrocatalytic pollutant degradation. The alloying effect of Pd and Ag modifies the surface electronic structure via strain and ligand effects, reducing the Pd noble-metal content while improving catalytic performance (see Figure 8).

### 3.2. Lead Sulfide (PbS)

Lead sulfide (PbS) is the primary mineral source of metallic lead, traditionally processed through high-temperature pyrometallurgy. However, conventional methods produce toxic SO_2_ and require energy-intensive conditions, raising environmental and occupational health concerns. A recent advancement demonstrates that PbS can be directly reduced electrochemically in DESs to produce metallic lead and elemental sulfur nanoparticles without the generation of SO_2_ [85].

Experiments employed a choline chloride-ethylene glycol (ChCl-EG) DES at 353 K, using a powder-cavity microelectrode filled with PbS powder. CV revealed a cathodic peak at −0.22 V (vs. Ag), corresponding to the one-step electro-desulfurization of PbS to Pb^0^ and S^2−^ ions. An anodic peak at −0.33 V was attributed to the dissolution of Pb, while a secondary oxidation at +0.35 V corresponded to S^2−^ → S^0^. This redox couple confirmed the viability of direct electrochemical desulfurization of PbS under near-room-temperature conditions. Electrolysis was conducted in a graphite crucible loaded with PbS powders (as the cathode) and a graphite plate as the anode, at applied voltages of 2.3–2.7 V for 3 h. Depending on the applied potential, different morphologies resulted. At 2.3 V, Pb formed as irregular nanoparticles (160–500 nm), whereas at 2.4–2.5 V, the morphology transitioned to coral-like Pb particles (1–2 μm). Finally, when the potential is increased to 2.6–2.7 V, mixed morphologies of aggregated, needle-like, and octahedral Pb grains emerged. Simultaneously, elemental sulfur nanoparticles (30–65 nm, amorphous, globular) were generated at the anode. Notably, no SO_2_ gas was detected, highlighting the green chemistry advantage of this DES-based approach. XRD confirmed complete conversion of PbS into metallic Pb at ≥2.4 V. TEM analysis of Pb grains revealed well-defined (111) lattice planes, consistent with γ-Pb crystallites. Fast Fourier Transform (FFT) analysis of sulfur confirmed its amorphous character, attributed to the relatively low electrolysis temperature (353 K). Thermodynamic analysis revealed that the theoretical decomposition potential of PbS (0.505 V at 353 K) is significantly lower than that of PbO (0.945 V), which explains why PbS reduction occurs at more positive potentials. This aligns with the observed onset potential (−0.22 V vs. Ag). This study establishes a proof-of-concept for green hydrometallurgical processing of sulfide ores in DESs.

### 3.3. Cobalt-Based Alloys: Pd-Co and Co-Smh

Pd-Co and Co-Sm alloys have been successfully synthesised in choline chloride-based eutectics, demonstrating advantages over conventional aqueous routes in terms of stability, composition control, and functional performance.

Pd-Co nanoparticles prepared from a mixture of ChCl and Urea illustrate the power of DESs to control nucleation and growth processes. Potentiodynamic and potentiostatic studies revealed the formation of multiple 3D nucleation and diffusion-controlled growth of bimetallic clusters [86]. Particle sizes could be tuned by adjusting the potential and deposition time, as confirmed by SEM and XPS, indicating the formation of an alloy. The resulting Pd-Co nanoparticles demonstrated enhanced activity toward the formic acid oxidation reaction (FAOR), outperforming Pd-only systems. Importantly, DES media enables clean co-deposition without stabilisers, thereby avoiding the limitations of aqueous electrolytes, where additives are typically required to prevent agglomeration.

Cojocaru et al. reported the electrodeposition of magnetic SmCo nanowires (SmCo NWs) from ChCl:Urea using nanoporous alumina templates. The electrolyte contained chlorinated salts of Sm and Co, and NWs with homogeneous thickness (~50 nm) were obtained by electrodeposition under galvanostatic conditions at current densities of −0.5 to 1 mA/cm^2^. The composition of the SmCo NWs was found to be 46–50 wt% Sm (around 2–2.5:1 Co:Sm ratio). The XRD analysis revealed the material’s crystallinity, identified a hexagonal phase, and showed good coercivities (350 Oe) [87].

### 3.4. Key Conclusions for Alloy Nanostructures

DESs provide a unique platform for electrochemical synthesis of alloy nanostructures, enabling co-deposition of multiple metals without stabilisers and thus overcoming the limitations of aqueous electrolytes. In addition, the high viscosity of DES prevents agglomeration of the resulting nanomaterials. The alloy composition can be adjusted by varying the precursor concentration in the bath or the applied potential. Therefore, uniform alloys with tuneable morphologies from nanoflowers (Au-Pt in ethaline) to porous and spiked nanostructures (Cu-Ag in ChCl:urea) and nanoparticles (Pd-Ag and Pd-Co in ChCl: EG or ChCl:urea) were obtained. The synthesised alloy nanostructures display superior electrocatalytic performance, particularly for the oxidation of formic acid, ethanol, and xanthene.

## 4. Non-Metallic Nanostructures

### Selenium Nanorods

Li et al. [88] prepared selenium nanorods by the solid-state electrolysis technique from Cu_2_Se and using ChCl:Urea (1:2) as electrolyte. During the synthesis, while copper metal is formed on the cathode, selenium nanoroads are obtained on the anode. At the cathode, Cu_2_Se forms an intermediate Cu_2−x_Se phase, which facilitates the reduction of Cu^+^ to metallic copper. Since selenium ions (Se^2−^) are gradually released into the electrolyte, they migrate to the anode, where they are oxidised to form selenium nanorods. The TEM analysis showed that the nanorods present a 1D morphology with a diameter of 157 nm and a length of 2.42 µm. The lattice spacing of 0.38 and 0.30 nm corresponded to the (100) and (101) phases of selenium, respectively.

## 5. Oxide Nanostructures

The formation of metal oxide nanoparticles in DESs typically follows one of two routes: the electrochemical pathway or solvothermal or sol–gel routes. Key advantages of electrochemical synthesis in DESs include lower synthesis temperatures, narrower size distributions, and surface functionalisation without the need for additional capping agents [59].

### 5.1. Iron Oxide (Fe_3_O_4_) Nanostructures

Magnetic iron oxide nanoparticles are widely investigated for biomedical and environmental applications, encompassing magnetic resonance imaging, drug delivery, biosensing, and remediation. A persistent challenge, however, lies in preparing highly dispersible, water-soluble nanoparticles with a narrow size distribution in a scalable and reproducible manner. Conventional co-precipitation routes produce polydisperse, poorly crystalline particles, while thermal decomposition provides better control but yields hydrophobic nanocrystals that require additional ligand exchange or surface coating.

A recent advance addressed these limitations by utilising DES electrolysis (DES-electrolysis), which enables the direct electrochemical synthesis of water-soluble magnetic iron oxides [89]. In this method, iron foils serve as both anode and cathode in a DES composed of choline chloride, choline phosphate, and urea. During electrolysis, iron atoms at the anode are oxidised to Fe^3+^ ions, which rapidly combine with reactive oxygen species generated by the decomposition of DES. This results in the nucleation of iron oxide nanocrystals (γ-Fe_2_O_3_). At the same time, decomposition of amine-containing DES components grafts hydrophilic amino groups directly onto the nanoparticle surface. Thus, water solubility and colloidal stability are achieved without any post-synthetic modification or stabilising ligands. The synthesised nanoparticles exhibit controlled size, as revealed by TEM, with monodisperse particles ranging in diameter from 6 to 9 nm. The hydrodynamic sizes in dispersion were 20–30 nm due to solvation. Amino group grafting was confirmed by FTIR and XPS, ensuring high surface charge and colloidal stability. Zeta potentials of +40–50 mV maintained transparent, precipitation-free dispersions for over 600 days. High-resolution TEM and FFT confirmed well-crystallised γ-Fe_2_O_3_ domains, correlating with strong magnetic behaviour. The nanoparticles retained bulk-like magnetic properties, with saturation magnetisation values of up to 72 emu g^−1^, comparable to those of high-purity γ-Fe_2_O_3_ powders. Hysteresis loops revealed ferrimagnetic behaviour with low coercivity, making it suitable for biomedical applications such as MRI contrast agents.

### 5.2. Zinc Oxide (ZnO) Nanoparticles

Zinc oxide (ZnO) is a wide-band-gap semiconductor (Eg ≈ 3.2 eV) with applications in photocatalysis, sensors, solar cells, and biomedical systems. DESs, particularly in emulsion and microemulsion, have emerged as promising green platforms for electrochemical ZnO synthesis [90].

In the emulsion-DES-assisted electrochemical synthesis approach, Zn^2+^ precursors were extracted from olive leaves, then subjected to electrochemical deposition in mixed DESs composed of ethaline (ChCl-ethylene glycol) and oxaline (ChCl-oxalic acid), in the presence of aniline as a polymer additive. A three-electrode electrochemical cell (Pt working electrode, Pt counter, Ag/AgCl reference) was used at room temperature. CV confirmed the formation of polyaniline (PANI), which served as a stabilising and conducting matrix for ZnO nanoparticles. The addition of aniline and small amounts of water was crucial to forming uniform ZnO-PANI nanocomposites. Compared with aqueous or simple DES systems, emulsion DESs provided larger interfacial areas, promoting controlled nucleation and limiting agglomeration of ZnO crystallites. SEM and TEM analyses revealed spherical ZnO nanoparticles with diameters ranging from 27 to 52 nm, depending on the DES mixture. Microemulsion systems yielded more homogeneous and finer distributions compared to aqueous analogues. XRD confirmed the wurtzite structure of ZnO, with crystallite sizes of ~37 nm, as calculated by the Scherrer equation. FTIR and UV-Vis spectra showed clear signatures of Zn-N and Zn-O-PANI bonding, indicating an intimate interaction between ZnO and polyaniline.

ZnO-PANI composites exhibited strong absorption in the UV region, with band-gap values of 2.98–3.06 eV, depending on the DES composition. Compared to ZnO prepared in aqueous solutions, DES-derived ZnO displayed slightly narrowed band gaps, attributed to modified surface states and polymer-semiconductor coupling. Electrochemical analysis revealed multiple redox peaks corresponding to the oxidation/reduction of PANI, accompanied by the formation of ZnO nanostructures. The incorporation of PANI enhanced the nanoparticles’ conductivity and dispersion.

Costovici et al. [91] reported the electrochemical synthesis of spherical ZnO nanopowder from a ChCl:urea electrolyte with zinc acetate. ZnO NPs were obtained through anodic dissolution of Zn metallic strips under direct current conditions at various current densities from 15 to 50 mA/cm^2^ and room temperature. The faradic current efficiency observed was high, around 90–95%. To minimise the deposition of Zn on the cathode, tetrabutylammonium bromide (TBAP) and poly(N-vinyl-pyrrolidone) were added to the electrolyte. After synthesis, the nano powder was calcined at 300 °C, yielding a crystalline hexagonal wurtzite structure with particle sizes ranging from 15 to 31 nm.

### 5.3. Titanium Dioxide (TiO_2_) Nanostructures

**Figure 9:**
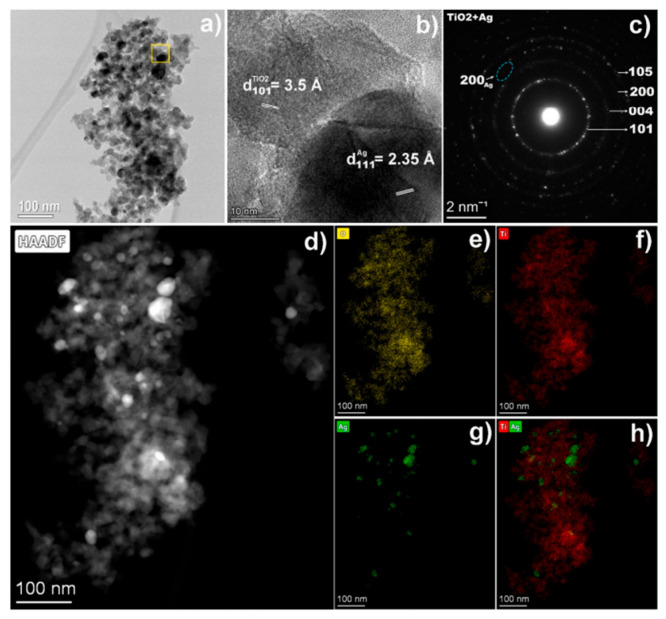
(**a**) TEM and (**b**) HRTEM image of Ag-TiO_2_–3 h composite, showing Ag particles on top of TiO_2_ (region marked with yellow square in (a)); (**c**) SAED pattern corresponding to the TEM image (**a**). (**d**) low-magnification HAADF-STEM image and EDS mapping showing the O (**e**), Ti (**f**) and Ag (**g**) distribution. (**h**) superimposed maps of Ti and Ag. Reproduced with permission of Pectu et al. [93]. © 2025 The Authors. Published by Elsevier B.V.

TiO_2_ nanopowders were synthesised by anodic dissolution of Ti metal in ChCl mixtures with ethylene glycol and urea. Apart from DES, the electrolyte contains ethanol and TBAB. The latest facilitates the continuous dissolution of Ti metal. In both solutions, high Faradaic efficiencies above 92% were observed for current densities ranging from 20 to 70 mA cm^−2^. After drying and calcination, both nanopowders exhibit crystallinity in their XRD patterns, with only the anatase phase being identified. According to SEM, the nanoparticles exhibit spherical morphologies with sizes ranging from 10 to 20 nm. The photocatalytic activity of the electrochemically synthesised TiO_2_ NPs was investigated towards the degradation of orange II dye solutions. Compared with commercially synthesised anatase TiO_2_, electrochemically synthesised TiO_2_ in DES showed higher photodegradation efficiency both under UV and visible irradiation [92]. Petcu et al. [93] reported the decoration of TiO_2_ nanopowders with Ag NPs using pulsed reverse current (PRC) electrodeposition in a ChCl:Ethylene glycol mixture. Various PRC parameters were varied, including the total electrodeposition time, which was extended from 1 h to 3 h, then to 6 h. The diffuse reflectance spectra (DRS) exhibited a broad band in the visible light region from 400 to 650 nm associated with the localised surface plasmon resonance (LSPR) of Ag NPs on the semiconductor’s surface. TEM analysis confirmed the presence of Ag NPs with sizes ranging between 10 and 35 nm decorating the TiO_2_ NPs (19.5 ± 10 nm) (see Figure 9). The composite was tested for the degradation of methyl orange dye under UV and visible light. The sample obtained after 3 h of total electrodeposition time showed the best performance, achieving 60.2% efficiency under visible light, compared with 32.3% for TiO_2_ NPs. The mechanism for dye degradation showed that the superoxide radicals play a crucial role. Furthermore, the antibacterial activity against E. coli and B. subtilis was clearly enhanced by incorporating Ag.

TiO_2_ nanotubular layers (TNT) were obtained by anodization in various types of DES based on ChCl with ethylene glycol (1:2), urea (1:2) and malonic acid (1:1). In some electrolytes, small amounts of NH_4_F from 0.2 to 0.75 wt.% were added. If the viscosity of DES is low, as is the case for ChCl:ethylene glycol mixtures, TNT was obtained at room temperature and 20 V, with interpore distances of 65 ± 5 nm, wall thickness of 10 ± 2 nm, and tube length of 2.6 ± 0.2 nm. However, for more viscous electrolytes, such as ChCl urea and ChCl:malonic acid, a higher temperature is required to enhance mass transport during anodization. In the malonic acid DES type, the interpore distance was the largest, at 110 ± 5 nm. Furthermore, the kind of electrolyte influences pore length, as it depends on the electrolyte’s viscosity. For example, 2.1 µm pores are formed from ChCl:malonic acid, while 2.6 µm pores are obtained from ChCl:ethylene glycol. The morphologies obtained in DES electrolytes are similar to those reported from aqueous electrolytes [94].

Chen et al. [95] reported the synthesis of TiO_2_ nanobamboos (TiO_2_ NBs) (nanotubes decorated with periodic exterior rings) by anodization from a fluoride aqueous electrolyte with the addition of 2–10% DES mixture of ChCl:succinic acid (1:1 molar ratio). When the anodization is performed with 5 wt.% DES and 20 V, TiO_2_ NBs with an average width of 80 nm and a thickness of bamboo rings of 10 nm were formed. Upon increasing the DES concentration in the electrolyte from 5 to 10%, the tube diameters increased from 65 to 100 nm, and their lengths also increased from 1.2 to 1.5 µm after 5 h of anodization. The authors proposed a mechanism involving various steps (I) rapid formation of tubular structures, followed by a (II) decrease in current density due to the accumulation of etching byproducts, (III) formation of dense oxide film, is evident as the rings on the NBs, and finally (IV) local dissolution of the dense oxide film, the dense oxide film does not cover the entire space among the tubular cells.

### 5.4. Key Conclusions for Oxide Nanostructures

Oxide nanostructures, including Fe_2_O_3_, ZnO, and TiO_2_, were successfully electrochemically synthesised in DES electrolytes. Some of them were obtained by anodic dissolution of iron foils, zinc metallic strips, or a titanium disc. In the case of magnetic nanoparticles, the iron oxidation and DES decomposition occur concurrently, producing monodisperse particles decorated with amino groups, ensuring long-term colloidal stability. Emulsion-assisted DES systems have also been effective for preparing ZnO-polyaniline composites, in which the presence of the polymer and microemulsion interfaces controls ZnO nucleation and limits agglomeration. Simple type III DESs such as ChCl:urea support anodic dissolution processes that yield ZnO and TiO_2_ nanopowders with high Faradaic efficiency and nanoscale crystallinity. In anodization processes, DES composition and viscosity govern the formation of TiO_2_ nanotubes and nanobamboo structures, influencing pore size, tube length and ring formation. The oxide nanostructures have been successfully used for the photodegradation of organic dyes and exhibit antibacterial properties, especially when decorated with Ag nanoparticles. DES media enable the synthesis of oxide nanostructures under mild conditions, with reasonable control over particle size, morphology, and surface chemistry, while avoiding many of the limitations associated with aqueous electrolytes.

## 6. Carbon-Based Nanostructure Composites

Composites synthesised in DESs through electrochemical protocol have attracted particular attention because they combine multiple components (carbon, metal oxides, alloys) into hybrid architectures that synergistically enhance catalytic, photocatalytic, and electrochemical properties. For instance, the use of DESs in preparing carbon-nanotube composites or carbon-metal oxide hybrids enables control over interfacial bonding, prevents agglomeration, and improves conductivity [59]. Similarly, metal-oxide composites prepared in DESs exhibit improved charge separation and defect engineering that are difficult to achieve in aqueous or organic solvents.

### 6.1. Carbon Nanotube-Based Composites: Ag-MWCNTs

**Figure 10:**
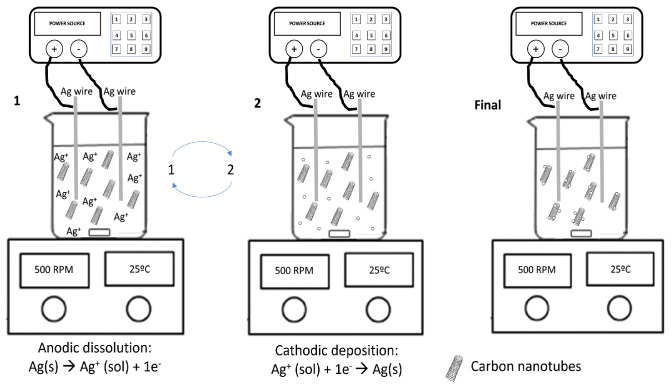
Schematic representation of the pulsed reverse current electrodeposition. Reproduced with permission of Brandão et al. [96]. © 2021 The Authors. Published by Elsevier B.V.

Several works highlight the electrodeposition of silver nanostructures onto multi-walled carbon nanotubes (MWCNTs) in DESs, as presented in Figure 10. Using choline chloride-glycerol mixtures (“glyceline”), Ag-MWCNT composites can be prepared by pulse-reverse electrodeposition, which enables a uniform nanoparticle decoration without the need for prior CNT functionalization [96]. Compared to aqueous systems, the DES medium reduces agglomeration, resulting in smaller and more uniformly distributed silver nanoparticles, and promotes stronger interfacial adhesion with the carbon framework. Electrochemical evaluation demonstrates that Ag-MWCNT electrodes prepared in DESs exhibit superior catalytic activity and stability for energy-related applications. For instance, composites obtained by pulse-reverse deposition achieved a specific capacitance of up to ~28.5 F g^−1^, a six-fold improvement over pristine MWCNTs, with excellent cycling retention (≈99% after 1000 cycles). These enhancements are attributed to the combined effects of Ag nanoparticles’ high conductivity and the stabilising role of the DES medium during nucleation and growth.

A key advance from these studies concerns the stability of dispersion. Bare MWCNTs are prone to bundling due to van der Waals interactions, resulting in poor colloidal behaviour. The decoration with Ag nanoparticles markedly improves dispersion by reducing surface energy and suppressing aggregation [97]. Dynamic light scattering (DLS) and UV-Vis analyses confirmed that Ag-MWCNT dispersions in glyceline remained up to 25× more stable than those of unmodified CNTs, with UV-Vis absorbance retention of ~97% after 120 h, compared to only ~50% in water [97]. Such stability improvements are crucial for reproducible processing and maximising the electrochemical surface area in practical devices.

Beyond electrochemical performance, silver decoration also influences the dispersions’ physicochemical properties. Measurements of viscosity, density, refractive index, surface tension, and ionic conductivity in glyceline revealed that Ag-MWCNTs consistently outperform pristine CNTs [98]. Notably, the incorporation of Ag nanoparticles shifted the zeta potential towards more negative values (down to −33 mV), indicating more stable dispersions in both water and DESs. A paradoxical but beneficial effect was observed in viscosity trends: dispersions of Ag-MWCNTs in glyceline exhibited a marked decrease in viscosity at lower temperatures, improving processability. These property shifts confirm that the synergy between metallic decoration and DES media tailors both interfacial interactions and bulk fluid behaviour.

### 6.2. Cobal Nanoclusters on Graphite Sheets

**Figure 11:**
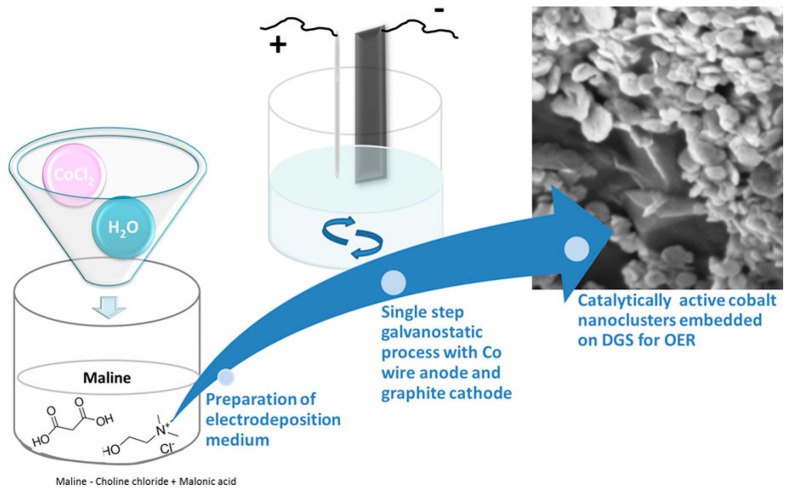
Schematic representation of the electrodeposition medium for cobalt NCs. Reproduced with permission of Renjith et al. [99]. Copyright © 2020. American Chemical Society.

The electrodeposition of the Co nanoclusters (Co NP) on graphite sheets was carried out in a single step using Co wire as anode and graphite substrate as cathode from a DES based on a mixture of ChCl:malonic acid (1:1) (see Figure 11). To decrease the viscosity of DES, 20% water was added to the mixture. The incorporation of water in the electrolyte facilitates the formation of well-separated nanoclusters of cobalt, with sizes ranging from 50 to 80 nm, on the surface of a dense graphite sheet. Without water, a film is observed on the graphite sheet. The material was used as a catalyst for the oxygen evolution reaction (OER). The CoNPs decorating graphite sheets synthesised in the presence of water exhibit superior catalytic activity compared to the material obtained just in DES. The effect was attributed to morphology, since many catalytically active edges are present due to the Co cluster formation. For the OER activity, the overpotential required to reach 10 mA·cm^−2^ was 50 mV [99].

### 6.3. Biomass-Derived Carbon-TiO_2_ Composites

The integration of titanium dioxide (TiO_2_) with carbon materials has emerged as a promising route to overcome the limitations of each component. Carbon offers high conductivity and surface area, while TiO_2_ provides redox activity, stability, and semiconducting character. A recent work has focused on combining these two materials within DESs, particularly ethaline (ChCl-ethylene glycol, 1:2), which provides a green, tunable medium for both synthesis and assembly.

Two main approaches have been reported for the synthesis of TiO_2_^−^ carbon composites using biomass-derived carbon matrices [100]. The ex situ physical attachment of TiO_2_ nanoparticles (commercial or DES-electrochemically synthesised) was achieved by dispersing them with glycogen-derived carbon in ethaline glycol, using ultrasound (sonication) to promote attachment. This yielded composites with moderately improved surface areas (up to ~1888 m^2^ g^−1^) compared to the pristine carbon (1526 m^2^ g^−1^). On the other hand, the in situ decoration during TiO_2_ synthesis. In this method, amorphous TiO_2_ nanoparticles were produced by electrochemical anodization of titanium in ethaline, with the carbon simultaneously dispersed in the electrolyte. This strategy resulted in uniform coverage of carbon sheets with TiO_2_, delivering the highest structural properties, with surface areas reaching 2214 m^2^ g^−1^ and enlarged pore diameters. These results show that the DES environment not only supports nanoparticle formation but also facilitates intimate interfacial contact between TiO_2_ and carbon, especially in in situ routes.

**Figure 12:**
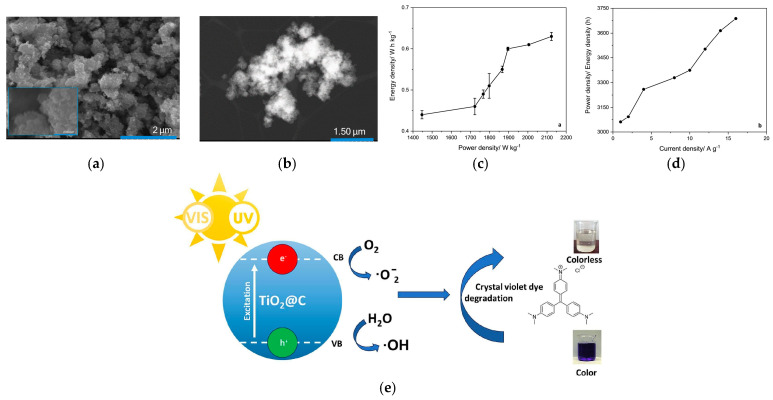
SEM and TEM micrographs (**a**,**b**), Ragone plot (**c**), Power density/Energy density vs. current density (**d**) and the schematic representation of the photocatalytic activity of the TiO_2_@C composite for application in energy storage and photocatalysis, (**e**) photocatalytic performance of the amorphous TiO_2_-decorated biocarbon composite for crystal violet dye degradation Refs. [100,101] Permissions: (**a**–**d**) © 2.024 The Authors. ChemSusChem published by Wiley-VCH GmbH (Creative Commons CC-BY-NC-ND), (**e**) © 2025 The Authors. Published by Elsevier Ltd. (Creative Commons CC-BY).

The electrochemical behaviour of TiO_2_-carbon composites (SEM and TEM presented in Figure 12a and Figure 12b, respectively) is strongly dependent on the attachment method and the amorphous/crystalline nature of TiO_2_. In a three-electrode configuration, the in situ TiO_2_@C composite achieved a capacitance of 956 F g^−1^ at 1 A g^−1^, with excellent retention (100% after 1000 cycles and 98% after 10,000 cycles). In a symmetric two-electrode solid-state device (ethaline-based electrolyte), the capacitance further increased to 1251 F g^−1^, retaining ~90% of its value after 1000 cycles. These results significantly outperform previously reported crystalline TiO_2_-carbon systems, highlighting the beneficial role of amorphous TiO_2_ combined with high-surface-area biomass-derived carbons.

A complementary study emphasised the photocatalytic activity of TiO_2_-carbon composites synthesised in DESs. Amorphous TiO_2_@C degraded up to 98% of crystal violet dye within 5 h under UV-Vis light, demonstrating strong applicability in wastewater remediation. The in situ decorated composite again showed superior performance compared to ex situ mixtures, confirming that close TiO_2_-carbon integration maximises both charge transfer and active site accessibility [101].

### 6.4. Key Conclusions for Carbon-Based Nanostructured Composites

DESs enable the controlled fabrication of carbon-based nanocomposites with enhanced dispersion stability, uniform nanoparticle decoration and improved functional performance. The physicochemical properties of DES allow the formation of Ag-MWCNT composites with uniformly distributed nanoparticles, reduced agglomeration, and improved electrochemical behaviour. DESs also stabilise carbon dispersion, thereby extending colloidal stability. Beyond CNT systems, DESs facilitate the controlled electrodeposition of Co nanoclusters onto graphite sheets and support the assembly of biomass-derived carbon-TiO_2_ composites through both ex situ and in situ formation routes, the latter yielding the highest surface areas and superior capacitive performance. As a result, carbon nanocomposites electrochemically synthesised in DESs exhibit enhanced catalytic, electrochemical, and photocatalytic activities, demonstrating that DESs are a powerful and versatile platform for engineering advanced carbon-based nanomaterials for energy storage, catalysis, and environmental applications.

## 7. Fundamental Interplay Between DES Physicochemical Properties and the Thermodynamics and Kinetics of Nucleation and Growth

The electrochemical formation of nanomaterials proceeds through two coupled stages: nucleation and growth, whose rates and pathways are governed by thermodynamics (nucleation barrier, interfacial energy, redox potentials) and kinetics (mass transport, charge transfer, adsorption). While numerous studies report that DESs enhance nucleation density and enable shape-controlled growth, a unified mechanistic explanation remains rare [57]. In DES-based electrochemical systems, the unique physicochemical environment profoundly modifies both the thermodynamic driving forces and the kinetic limitations of nanostructure formation.

From a thermodynamic perspective, the low surface tension of DESs reduces the solid–liquid interfacial energy (γ), directly lowering the classical nucleation barrier (ΔG* ∝ γ^3^/Δμ^2^) [102]. As a result, DESs promote high nucleation densities even at modest overpotentials. Simultaneously, the strong hydrogen-bond network of DESs stabilises complexed metal species, shifting redox potentials and altering supersaturation levels. Metal ions frequently exist as chloride-, urea-, or polyol-coordinated complexes, thereby modifying their reduction thermodynamics relative to those in aqueous systems. This DES-dependent speciation partially explains the broader range of accessible morphologies and the surfactant-free shape control observed for noble and transition metals.

Kinetic effects arising from DES viscosity and ionic organisation further dictate particle evolution [16]. The high viscosity of most DESs decreases diffusion coefficients by up to two orders of magnitude relative to water, slowing the supply of electroactive species to growing nuclei. This shift favours diffusion-limited growth, suppresses coalescence, and stabilises small nuclei that would otherwise dissolve or aggregate. At high overpotentials, however, the wide electrochemical window of DESs enables rapid reduction kinetics, creating conditions in which nucleation becomes nearly instantaneous, producing ultra-high densities of small seeds that subsequently grow slowly due to mass-transport constraints.

The high ionic strength and the strong structural effects inherent to DESs impose additional scale effects [103]. Ion pairing and HBD/HBA interactions alter double-layer structure, local electric fields, and adsorption dynamics on the electrode surface. These factors influence facet-selective growth by stabilising or blocking specific crystallographic planes. For example, urea adsorption in ChCl:urea selectively suppresses growth on low-index facets, enabling the formation of concave polyhedral structures in Pt and Pd. Such selective adsorption is analogous to surfactant-directed growth but arises intrinsically from DES composition.

Temperature plays a dual kinetic role: increasing temperature rapidly decreases viscosity, enhancing diffusion, while also accelerating DES self-decomposition pathways that may generate adsorbates or intermediates participating in growth. Consequently, transitions between progressive nucleation, instantaneous nucleation, and mixed regimes frequently occur as a function of temperature or applied overpotential.

In combination, these thermodynamic and kinetic features establish a DES-specific nucleation and growth landscape characterised by: (i) lowered nucleation barriers; (ii) high and spatially uniform nucleation densities; (iii) slow, diffusion-limited growth; and (iv) selective facet stabilisation without external surfactants.

Although DESs share standard physicochemical features, the specific identity and ratio of the hydrogen-bond acceptor (HBA) and hydrogen-bond donor (HBD) impart substantial variations in solvation structure, ion speciation, viscosity, conductivity, and complexation strength. These differences profoundly affect nucleation and growth behaviour, yet a systematic comparison across DES families remains scarce. The majority of studies rely on a narrow subset of Type III DESs—most commonly reline (ChCl:urea) and ethaline (ChCl:ethylene glycol)—and generalising their behaviour to the broader DES landscape is not always justified.

Comparing results across DES families reveals several emerging design principles. Reline, characterised by strong hydrogen bonding and relatively high viscosity, promotes slow diffusion and high nucleation densities, which favour the formation of small nuclei and stabilise complex morphologies such as nanoflowers, concave polyhedra, and branched structures. The strong coordinating ability of urea can also lead to facet-selective adsorption, enabling shape control without the need for surfactants. In contrast, ethaline exhibits lower viscosity, higher ionic conductivity, and weaker coordination, leading to faster mass transport, larger particle sizes, and morphologies that are more sensitive to applied potential than to ligand-directed growth. Ethylene glycol–based DESs also stabilise intermediate metal–glycolate adducts, altering the reduction thermodynamics relative to urea-based systems. These compositional effects highlight that DES chemistry directly dictates nucleation pathways and morphology evolution rather than simply providing an inert solvent matrix.

Beyond Type III systems, studies involving Type I/II (metal salt–based) DESs and acidic Type IV DESs demonstrate enhanced metal–ligand interactions, generating highly reactive metal–halide or metal–oxide complexes that shift redox potentials and promote rapid nucleation. However, systematic comparisons are still missing. Even more underexplored are Type V DESs (non-ionic HBA/HBD combinations) and Natural Deep Eutectic Solvents (NADES). Their intrinsically lower viscosity, higher hydrophobic tunability, and biocompatible components offer unique opportunities: (i) enabling low-temperature, additive-free anisotropic growth; (ii) reducing aggregation due to weaker salt structuring; (iii) providing environmentally benign routes for biomedical or sensing applications; and (iv) producing reactive organic radicals or intermediates that may participate in nanoparticle nucleation.

The current literature thus remains heavily skewed toward classical choline chloride–based Type III DESs, while the broader chemical diversity of DES families is far from fully explored. Expanding the field to include Type V DESs, NADES, and non-choline-based systems will allow the rational design of DES environments tailored to specific nucleation barriers, reduction pathways, and facet-selective growth mechanisms. Such diversification presents a significant opportunity to shift from empirical solvent selection to predictive, composition-driven design principles for electrochemical nanomaterial synthesis

## 8. Conclusions and Future Perspectives

This review highlights recent advances in the development of metal, alloy, oxide, and carbon-based composite nanomaterials obtained by electrochemical routes from DESs, along with their applications.

One of the significant advantages of using DESs in the electrochemical synthesis of nanomaterials lies in their sustainability and environmental compatibility. Type III DESs and NADES are particularly sustainable due to the recyclability of choline chloride and the use of naturally derived components. These solvents can be easily recovered and reused, minimising chemical waste and reducing the environmental footprint of the synthesis process. Derived from renewable and biodegradable sources, DESs offer a green alternative to conventional organic solvents and ionic liquids, which often exhibit higher toxicity and poor biodegradability. The choice of individual DES components strongly influences their biodegradability and toxicity profile. Additionally, DESs exhibit low volatility, which minimises harmful emissions and worker health risk, and their low melting points contribute to energy-efficient processing [35]. From an electrochemical perspective, DESs provide high electrochemical stability, excellent solubility for a wide range of precursors, low toxicity and a broad electrochemical window. Moreover, DESs possess low surface tensions, which promote high nucleation rates, and their high ionic strength further induces structural effects such as templating, capping, and stabilisation, which play crucial roles in controlling particle morphology and size distribution and preventing agglomeration. These features make DESs highly effective and sustainable media for the synthesis of nanomaterials with controlled morphologies.

A broad spectrum of nanomaterials has been prepared in DESs using electrochemical routes such as electrodeposition (pulsed electrodeposition, square-wave potential sequence), cyclic voltammetry (CV), chronoamperometry (CA), solid-state electrolysis or anodization. Metallic nanostructures, such as Au, Ag, Pt, and Pd, have been shaped into various geometries, ranging from spherical particles to more complex morphologies, including nanoflowers and concave tetrahexahedral and concave disdyakis triacontahedral structures, by adjusting the electrochemical parameters, such as applied potential and electrolyte temperature. Even nanowires (Bi NWs) were obtained by galvanostatic electrodeposition. Similarly, bimetallic systems, such as Pd-Ag, Au-Pt, Cu-Au, and Cu-Ag, have been electrodeposited into nanocrystalline or porous structures. By varying the electrochemical parameters, a tunable alloy composition was obtained. Oxide-based nanomaterials, such as TiO_2_ and ZnO, have also been obtained by anodic dissolution of titanium or zinc metal strips in DESs. Complex shapes such as TiO_2_ nanobamboos (NBs) were obtained by electrochemical anodization of Ti foil in DES-aqueous mixtures. Finally, composites such as TiO_2_-biocarbon, Ag-MWCNTs, or Co nanoclusters on graphite sheets were prepared using either in situ or ex situ methods. A comprehensive summary of the nanostructured materials electrochemically synthesised in various DES systems, together with their synthesis parameters, main properties, and reported applications, is presented in Table 2.

These DES-derived nanomaterials have been used in various applications. Metallic nanostructures, such as Au and Ag, have been utilised for sensing applications to detect DNA or nitrofurazone. In contrast, Pt and Pd nanostructures have demonstrated superior electrocatalytic activity for the oxidation of ethanol, methanol, and formic acid. Additionally, their alloys (e.g., Pt-Ag or Pt-Au) were utilised in the oxidation of formic acid and in catalytic xanthene oxidation. Metal oxides and their composites with carbon materials have proven highly effective in environmental remediations, achieving high degradation efficiencies for organic pollutants and dyes. In addition to their catalytic and environmental applications, DES-derived nanostructures have recently attracted significant attention in energy storage, particularly in supercapacitors. The electrochemical synthesis of carbon-based and hybrid materials such as TiO_2_–carbon composites, Ag–MWCNTs, and Co-decorated graphite sheets in DESs has enabled the fabrication of electrodes with high surface area, excellent conductivity, and long-term cycling stability. The unique ionic environment of DESs promotes uniform nucleation and defect engineering, yielding nanostructures with enhanced ion accessibility and charge transport pathways. For instance, in situ DES-mediated synthesis of TiO_2_–biocarbon electrodes has achieved specific capacitances exceeding 1000 F g^−1^ with outstanding retention, underscoring the potential of these green solvents to bridge high performance and sustainability.

A critical comparison of performance across applications shows that many DES-derived nanomaterials exhibit superior catalytic, sensing, and energy-storage behaviour because DES environments introduce structural and surface features that are difficult to obtain through conventional aqueous or organic syntheses. The high nucleation densities and slow, diffusion-limited growth inherent to viscous DES media often yield nanostructures with abundant high-index facets, defect-rich surfaces, and uniform size distributions. These characteristics directly enhance activity in electrocatalytic reactions such as ethanol, methanol, and formic acid oxidation, where the density of undercoordinated sites governs turnover frequencies. Likewise, the strong coordination and templating effects of HBD/HBA components can generate surface terminations not accessible in water—such as urea-stabilised concave Pt and Pd geometries—which exhibit higher intrinsic activity and stability than their conventionally synthesised counterparts. In sensing applications, DES-derived noble-metal nanostructures often provide larger electroactive surface areas and improved electron-transfer kinetics, enabling lower detection limits than nanoparticles grown in aqueous media, where coalescence and surfactant residues suppress active surface exposure. In contrast, DES-derived materials can underperform when the same physicochemical features impose transport or accessibility penalties. For example, incomplete removal of DES residues or strong ligand binding may block active sites, reduce conductivity, or inhibit mass transport in porous frameworks. Furthermore, DES systems with extremely high viscosities can yield deposits with limited long-range connectivity, resulting in lower performance in applications that rely on rapid ion/electron transport (e.g., high-rate supercapacitors). Comparative studies indicate that while oxide and hybrid materials synthesised in DESs often show superior defect engineering, surface functionalization, and dispersion stability, materials prepared in aqueous or solvothermal systems may outperform them in applications that require crystalline ordering or fast ion mobility. Overall, the functional performance of DES-derived nanomaterials is best understood as a consequence of their distinctive nucleation–growth regimes and the chemical participation of DES components, which together govern facet exposure, defect density, and interfacial chemistry in ways not achievable by traditional solvent systems.

Despite the significant progress achieved in recent years, several limitations still constrain the broader development and practical implementation of electrochemical nanomaterial synthesis in DESs. Most of the reported studies rely heavily on Type III DESs, which are composed of choline chloride mixed with urea, ethylene glycol, or glycerol. While these systems are cost-effective, biodegradable, and their properties can be easily tuned (hydrophobicity/hydrophilicity, viscosity, etc.), they are well-studied; their dominance in the literature limits the understanding of the vast chemical diversity of DESs [104]. Several other DES families remain insufficiently explored, largely due to technical or chemical constraints that have discouraged systematic investigations. Type I DESs, which incorporate anhydrous metal salts, are underutilised due to moisture sensitivity and handling difficulties [105]. Furthermore, types I, II, and IV DESs all contain metal salts, which inherently increase toxicity and raise environmental concerns [57]. Within type III DESs, systems based on organic acids (such as oxalic, acetic, citric, etc.) are much less used for nanomaterial synthesis, due to their corrosive nature, which may dissolve the forming nanostructures [106]. Future work should focus on exploring alternative HBDs and HBAs to tailor DES properties toward specific nanomaterial systems. Hydrophobic DESs, such as thymol- or menthol-based mixtures, have received limited attention due to limited characterisation. Nevertheless, they provide nonpolar environments, which are useful for the synthesis of hydrophobic nanomaterials. Looking ahead, machine learning offers a powerful opportunity to accelerate nanomaterial synthesis by predicting optimal HBD/HBA combinations guiding the rational selection of DES formulations [29].

Regarding the mechanistic understanding, electrochemical nucleation and growth in DESs follow consistent trends, shaped by the high viscosity, strong solvation, and specific adsorption behaviour of HBD species. Most studies report 3D nucleation with diffusion-controlled growth as shown for Ag, Pd, Cu, Fe, Pd-Ag and Pd-Co nanostructures, where current-time transients confirm classical nucleation models adapted to the slow ion transport characteristic of DES. Variation in the electrolyte temperature induces changes in the nucleation process (e.g., from progressive to instantaneous nucleation). In situ grazing-incidence small-angle X-ray scattering studies reveal that nanoparticle formation in DES proceeds through slow aggregation and the gradual depletion of metal ions near the electrode, processes that are far slower than in aqueous electrolytes due to suppressed diffusion. The role of the DES components is also evident: urea and other HBDs adsorb selectively on metal facets, directing the resulting morphologies, whereas residual water participates in parallel reduction pathways, generating thin hydroxide shells on metals. Despite overall agreement on diffusion-limited nucleation, the literature remains divided on the exact influence of water content, which may either accelerate nucleation or induce oxide formation depending on the metal and potential. Similarly, studies on the influence of HBD type on anisotropic growth remain limited, highlighting the need for more systematic in situ investigations. Although DESs clearly influence nanoparticle morphology and surface chemistry, the fundamental interactions between solvent components, metal ions and growing nuclei remain insufficiently characterised.

From an application perspective, most studies have tested nanomaterials synthesised in DES under ideal or simplified laboratory conditions. For instance, photocatalytic performance is often evaluated using model dye pollutants (e.g., methyl orange or methylene blue), which do not reflect the complexity of real water matrices. Extending photocatalytic studies to real wastewater samples containing a mixture of organic and inorganic contaminants (e.g., pharmaceuticals, pesticides, heavy metals) is crucial for validating the practical applicability of this approach. Finally, scalability also remains a significant bottleneck. Nearly all electrochemical synthesis in DESs is performed at the millilitre scale, which is adequate for fundamental research but insufficient for industrial applications. The high viscosity of DESs may be challenging during upscaling. The high viscosity of most DESs significantly complicates handling, transport, and agitation, increasing energy consumption and requiring oversized equipment compared to aqueous systems. Due to their high viscosity, low deposition rates are observed in DESs, and the electroactive species exhibit significantly lower diffusion coefficients. Even though DESs are often described as stable at elevated temperatures, many of them exhibit decomposition at unexpectedly low temperatures (e.g., a ChCl-ethylene glycol mixture near 90 °C), limiting the common strategy of increasing temperature to improve fluidity and transport properties. Moreover, DES electrolytes exhibit complex speciation, which strongly influences the electrochemical behaviour and is not yet fully understood, unlike in aqueous systems. Finally, although DESs are far less expensive than IL, their current cost (~40 $ per kg at 50–1000 kg scale) remains significantly higher than that of the acids and salts used in conventional aqueous electrolytes, posing an additional barrier to adoption at larger scale [107,108].

**Table 2:** Summary of the nanostructured materials electrochemically synthesised in DESs.

System	DES Type	Electrochemical Parameters	Properties and Applications	Ref.
Metal-based nanostructures				
Au nanoflowers	ChCl-Urea (1:2)	Cyclic voltammetry on GC electrode	The density of AuNFs on GC increases with the temperature of the electrolyte. The AuNFs-modified electrode was used as sensor for DNA detection (*M. Tuberculosis*)	[61]
Au nanocrystals	ChCl-Urea (1:2)	Electrodeposition on GC electrode at different potentials from −0.5 V to −0.95 V	Au NCs with different morphologies based on the applied potential from concave rhombic dodecahedra to concave cubes, octopods, cuboctahedral boxes, and finally, to hollow octahedra with application in D-glucose electrooxidation	[62]
Ag NPs	ChCl- Ethylene glycol (1:2)	Pulsed electrodeposition from 0.01 M AgCl at −0.5 V (vs. Ag/AgCl)	In situ approach describing the size and structural evolution of Ag NPs after pulse electrodeposition	[109]
	ChCl-Urea-Glycerol (1:1:1)	Electrodeposition at peak potential	The incorporation of SDS surfactant into DES led to a homogeneous Ag particle distribution. The Ag-SDS-modified electrodes have a wide linear range and low LOD for nitrofurazone detection	[64]
Au and Ag NPs on TiO_2_ nanotubes	ChCl-Urea (1:2)	Electrodeposition	Au and Ag NPs quasi-spherical with average size of 100 nm, uniformly distributed on TiO_2_ nanotubes	[65]
Pt nanoflowers	ChCl-Urea (1:2)	Cyclic voltammetry and constant potential deposition	Pt NFs with average size of 200 nm with enhanced electrocatalytic activity for ethanol oxidation	[66]
Concave tetrahexahedral Pt nanocrystals (THH Pt NCs	ChCl-Urea (1:2)	Electrodeposition using a square-wave potential sequence	THH Pt NCs with sized range from 62.5 nm up to 370 nm with enhanced electrocatalytic activity for ethanol oxidation	[67]
Pd concave-disdyakis triacontahedral palladium nanocrystals (C-DTH Pd NCs)	ChCl-Urea (1:2)	Electrodeposition using a square-wave potential sequence	C-DTH Pd NCs electrochemically shape controlled with enhanced electrocatalytic activity for ethanol electrooxidation	[68]
Pd NPs	ChCl:urea (1:2)	Electrodeposition	In situ approach showing that DES stabilises the electrodeposited Pd NPs	[69]
	ChCl:urea (1:2)	Electrodeposition on GC at two overpotentials	The deposition followed a 3D nucleation and diffusion growth with simultaneous reduction of water. PdNPs with Pd^0^ core and Pd(OH)_2_ shell. The material showed good electrocatalytic activity for methanol and formic acid oxidation reaction	[70,71,72]
	ChCl:Ethylen glycol (1:2)	Electrodeposition on GC	Uniform Pd NPs with sized of ~41 nm uniformly distributed on GC with activity for formic acid electrochemical oxidation	[73]
Ni NPs	ChCl:urea (1:2)	Electrodeposition on GC under potentiostatic conditions	Uniformly distributed Ni NPs with nickel metallic core and a thin layer of Ni(OH)_2_	[74,75]
Cu NPs	ChCl:urea (1:2)	Electrodeposition under constant potential on stainless steel from Cu_2_O precursor	The size of the Cu NPs depends on the applied potential, at 2.5 V, Cu NPs with 28 ± 7 nm are formed and at 2.2 V, 57 ± 6 nm	[76]
	ChCl:urea (1:2)	Electrodeposition under constant potential on nickel foil from Cu_2_O precursor	Cu NPs with narrow size distribution are obtained at lower temperature (303 K) and large overpotential (−0.80 V).	[77]
	ChCl:urea (1:2)	CV and CA studies from CuCl_2_·2H_2_O precursor	The mechanism for Cu NPs includes two contributions: 3D nucleation and diffusion-controlled growth + adsorption	[78]
Fe NPs	ChCl:urea (1:2)	CV and CA studies on HOPG	Fe NPs with cauliflower morphology and average size of 60 ± 8 nm. Core of Fe(0) and shell a mixture of FeO, Fe_2_O_3_ and Fe(OH)_3_	[80]
Bi NWs	ChCl: oxalic acid (1:1)	Galvanostatic electrodeposition under DC on Cu substrate	Bi NWs with diameters of 100–120 nm and a 1–1.5 µm length were obtained and used for H_2_O_2_ detection	[81]
Alloys nanostructures				
PbS	ChCl-Ethylene glycol (1:2)	Electroreduction of PbS powders at 2.3–2.7 V, 353 K	Pb nanoparticles (160 nm–2 μm, tunable morphology); S NPs (30–65 nm); no SO_2_ emission; green metallurgy	[85]
Au-Pt nanoflowers	ChCl-Ethylene glycol (1:2) + 10% H_2_O	Electrodeposition at −0.3 V, 30 °C	Nanoflowers (~500 nm); catalytic xanthene oxidation; electroorganic synthesis	[82]
Cu-Ag NPs	ChCl-urea (1:2)	Co-deposition at −0.65 to −0.75 V	Porous/spiked nanostructures (200–500 nm); tunable Cu:Ag ratio; high roughness factor; CO_2_RR, ORR	[83].
Pd-Ag NPs	ChCl-Ethylene glycol (1:2)	Electrodeposition on GCE and GCE/Ppy; CV, CA	50–60 nm alloy NPs; high activity for formic acid oxidation; reduced Pd usage; fuel cells	[84]
Pd-Co NPs	ChCl-urea (1:2)	Potentiostatic and potentiodynamic studies	3D nucleation; alloy NPs; enhanced formic acid oxidation vs. Pd; clean DES synthesis	[86]
Sm-Co NWs	ChCl-Urea (1:2)	Galvanostatic electrodeposition on nanoporous alumina templates	Uniform SmCo NWs of 50 nm diameters with hexanonal phase and higher coercivity (350 Oe)	[87]
Non-metallic nanostructures				
Se nanoroads	ChCl-Urea (1:2)	Solid-state electrolysis route	Nanoroads with 157 nm diameters and 2.42 µm length	[88]
Oxide nanostructures				
Fe_3_O_4_ NPs	ChCl-Choline phosphate-Urea	Electrolysis of Fe electrodes in DES; radical-assisted nucleation	6–9 nm γ-Fe_2_O_3_ NPs; amino-functionalized, stable > 600 days; magnetization 72 emu/g; MRI, drug delivery	[89]
ZnO NPs	ChCl-EG and ChCl-oxalic acid (emulsion DES)	Electrodeposition with aniline additive; CV and CA studies	27–52 nm ZnO; bandgap 2.98–3.06 eV; improved dispersion; photocatalysis and sensing	[90]
	ChCl: Urea	Anodic dissolution of Zn metallic strips	Spherical nanoparticles with sizes between 15 and 31 nm and crystalline hexagonal wurtzite structure	[91]
TiO_2_ NPs	ChCl: Ethylene glycol and ChCl: Urea	Anodic dissolution of Ti metallic strip	Anatase TiO_2_ nanoparticles with diameters between 10 and 20 nm with application in the photodegradation of methyl orange II dye (95.5% efficiency in UV light)	[92]
Ag-TiO_2_ NPs	ChCl: Ethylene glycol (1:2)	Pulsed reverse current electrodeposition	Spherical Ag NPs with sizes 10–35 nm distributed on agglomerated TiO_2_ NPs, photodegradation efficiency for methyle orange dye (60.2 under visible light) and antibacterial activity against *E. coli* and *B. subtilis*	[93]
TiO_2_ nanotubular layers (TNT)	ChCl mixtures with ethylene glycol (1:2), urea (1:2) and malonic acid 1:1)	Electrochemical anodization of titanium plates in DES with NH_4_F (0.2–0.75 wt.%) and water (1.79 wt%)	Nanotubes with interpore distance between 65 and 110 nm, and wall thickness of 10 nm. The pore length depends on the electrolyte viscosities (2.1 to 2.6 µm)	[94]
TiO_2_ nanobamboos (NBs)	ChCl: succinic acid (1:1)	Electrochemical anodization of titanium foil in aqueous NH_4_F electrolyte with 2–10% DES	Nanotubes decorated with periodic exterior rings (NBs) with diameters of 40/65 nm (inner/outer), and rings width of 80 nm and thickness of 10 nm	[95]
Carbon-based nanostructure composites				
Ag-MWCNT	ChCl-Glycerol (glyceline)	Pulse-reverse electrodeposition, Ag wire; CV, CA studies	Uniform Ag NP decoration; improved dispersion stability (25× vs. CNTs); capacitance 28.5 F/g; stable sensing and supercapacitor electrodes	[96]
Co nanoclusters on graphite sheets	ChCl: malonic acid (1:1) + water (20%)	Single-step galvanostatic electrodeposition with Co wire anode and graphite cathode	The presence of water in DES facilitates the formation of Co nanoparticle cluster from 20 to 90 nm; application in the oxygen evolution reaction (350 mV overpotential)	[99]
TiO_2_-biomass-derived carbon	ChCl-Ethylene glycol (ethaline)	Anodization of Ti in DES; in situ vs. ex situ decoration on glycogen-derived carbon	High surface area (2214 m^2^/g); capacitance up to 1251 F/g; 98% dye degradation; supercapacitors and photocatalysis	[100]
	ChCl: Urea	Anodic dissolution of Zn metallic strips	Spherical nanoparticles with sizes between 15 and 31 nm and crystalline hexagonal wurtzite structure	[91]

A key direction for advancing DES-based nanomaterial synthesis is integrating modern characterisation and design methodologies to address current limitations. In situ and operando electrochemical techniques such as X-ray scattering, Raman spectroscopy, and electrochemical mass spectrometry are essential for resolving nucleation pathways, ligand dynamics, and DES–metal interactions in real time. Machine-learning and computational screening frameworks offer powerful routes for rational DES design by predicting optimal HBA/HBD combinations, viscosities, and coordination chemistries tailored to specific nanostructures or catalytic functions. In parallel, life-cycle assessment (LCA) and techno-economic analyses are needed to quantify the actual sustainability benefits of DES systems relative to conventional solvents. Finally, developing scalable flow-electrolysis reactors for DES media will be crucial for transitioning laboratory-scale syntheses toward industrial production while ensuring consistent mass transport and morphology control. These forward-looking strategies collectively outline a roadmap for overcoming current barriers and fully exploiting the potential of DESs in nanomaterial synthesis and electrocatalysis.

Overall, electrochemical synthesis in DESs stands at the intersection of green chemistry and advanced materials engineering. By combining tuneable solvent chemistry, morphology control, and environmental compatibility, DESs open new opportunities for scalable, eco-efficient nanomaterial production. The progress reviewed here confirms that DESs are more than sustainable alternatives; they are enabling platforms for next-generation functional materials in sensors, catalysis, energy conversion, and storage. With continued mechanistic understanding, the expansion of DES chemistries, and integration into real-world systems, DES-assisted electrochemical synthesis is poised to play a pivotal role in building a sustainable, electrified future.

## Figures and Tables

**Table 1:** Comparison of DESs with ionic liquids and water [29,30,31,32].

Property	Water	Ionic Liquids	Deep Eutectic Solvents
Tunability	Limited	Unlimited range of cation-anion combinations	Unlimited range of HBA-HBD combinations
Solvation	Limited (polar species)	Strongly solvating	Broad solvation spectrum
Cost	Low	2–100× cost of organic solvents	Less expensive than IL
Environmental concerns	Low	Often hazardous and non-biodegradable	Less toxic and more biodegradable
Polarity	Limited polarity variability	Moderate	Highly adjustable (hydrophilic → hydrophobic)
Catalytic ability	Limited	Common and tunable	Intrinsic
Thermal stability	Boiling point at 373 K	High (stable up to 673 K)	High (stable up to 473 K)
Melting point (K)	273	<373	<343
Density at 293 K (g/cm^3^)	0.9982	1.05–1.64	1.1–1.3
Viscosity at 298 K (cp)	0.89	10–726	5–1000
Vapour pressure at 298 K (Pa)	3.17 × 10^3^	<1	<10
Surface tension at 298 K (mN/m)	71.99	30–60	35–75
Specific heat capacity (J/mol K)	75.3	400–1500	180–700
Thermal conductivity (W/m K)	0.598	0.1–0.2	0.15–0.25
Ionic conductivity (mS/cm)	0.2	Usually, <10	1.5 to over 100
Electrochemical window (V)	1.23	>3.5	2–3.5

## Data Availability

No new data were created or analyzed in this study.
